# Case report of ureter obturator hernia and literature review analysis

**DOI:** 10.1186/s12894-021-00851-2

**Published:** 2021-05-29

**Authors:** Yongqiang Hong, Siyu Zhang, Xiuying Kong, Yuxin Zhang, Shaokun Hong, Yuedong Chen

**Affiliations:** 1 Department of Clinical Medicine, Fujian Medical University, Fuzhou, 350000 China; 2 Department of General Surgery, The First Affiliated Hospital of Xiamen niversity, Xiamen, 361001 China; 3 Xiamen University School of Medicine, Xiamen, 361001 China; 4 Department of Urology, The First Affiliated Hospital of Xiamen University, Xiamen, 361001 China

**Keywords:** Ureteral obturator hernia, Ureteral stent implantation, Hernia repair, Hydronephrosis, Case report

## Abstract

**Background:**

Ureteral obturator hernia is a rare condition, usually found accidentally during imaging examinations, or found during surgery. Ureteral hernia can easily lead to ureteral obstruction and hydronephrosis. Long-term hydronephrosis may lead to kidney damage and infection, and eventually cause kidney failure. As of December 31, 2020, there are only 2 literature reports.

**Case presentation:**

This article reports a 67-year-old female patient with no symptoms. The computed tomography (CT) scan of the urinary system to show the left kidney and ureter had hydrops. The CTU imaging of the urinary tract revealed the left ureter pelvis herniated into the parietal pelvic fascia was accompanied by tortuosity and left hydronephrosis. She underwent laparoscopic abdominal wall hernia repair on April 29, 2020, and she recovered well.

**Conclusions:**

Ureteral obturator hernia is an uncommon condition. The clinical symptoms are non-specific, including unclear abdominal pain, until the appearance of obstructive diseases of the urinary tract, such as renal insufficiency, urinary tract infection, kidney stones, and uremia. A comprehensive review of the literature shows that it is difficult to make an accurate diagnosis based on physical examination alone.Early urography can improve the possibility of accurate diagnosis. When a patient suffers from impaired renal function, timely surgical treatment can avoid deterioration of renal function.

## Background

Ureteral obturator hernia a rare occurrence, usually found accidentally during imaging examinations or during surgery [1, 2]. Ureteral obturator hernia belongs to a category of ureteral hernia. Ureteral hernia can occur in the groin, femoral ring, ischial foramen, obturator and chest [3]. Among them, ureteral hernia is extremely rare in the obturator position. The obturator is composed of the pubic bone and ischial branch. Most of the holes are occupied by a layer of membrane, with small holes on the caudal side to allow the passage of obturator veins, arteries and nerves [4]. When the abdominal organs protrude through the obturator foramen of the hip to the femoral triangle (composed of the inguinal ligament, the inner edge of the adductor longus muscle, and the inner edge of the sartorius muscle), it is called an obturator hernia. Obturator hernia is a rare type of abdominal wall hernia with an incidence rate of 1 % [5]. Once the ureter protrudes through the defect of the obturator foramen and cannot exit after entering the obturator tube, the ureteral hernia will occur into the obturator fora. We call it a “Ureteral obturator hernia”. Through searching historical documents, we found that there are only 2 reported cases of obturator hernia with ureteral compression. Here, we provide an interesting case of an elderly woman with obturator hernia and left ureteral compression.

## Case presentation

**Fig. 1:**
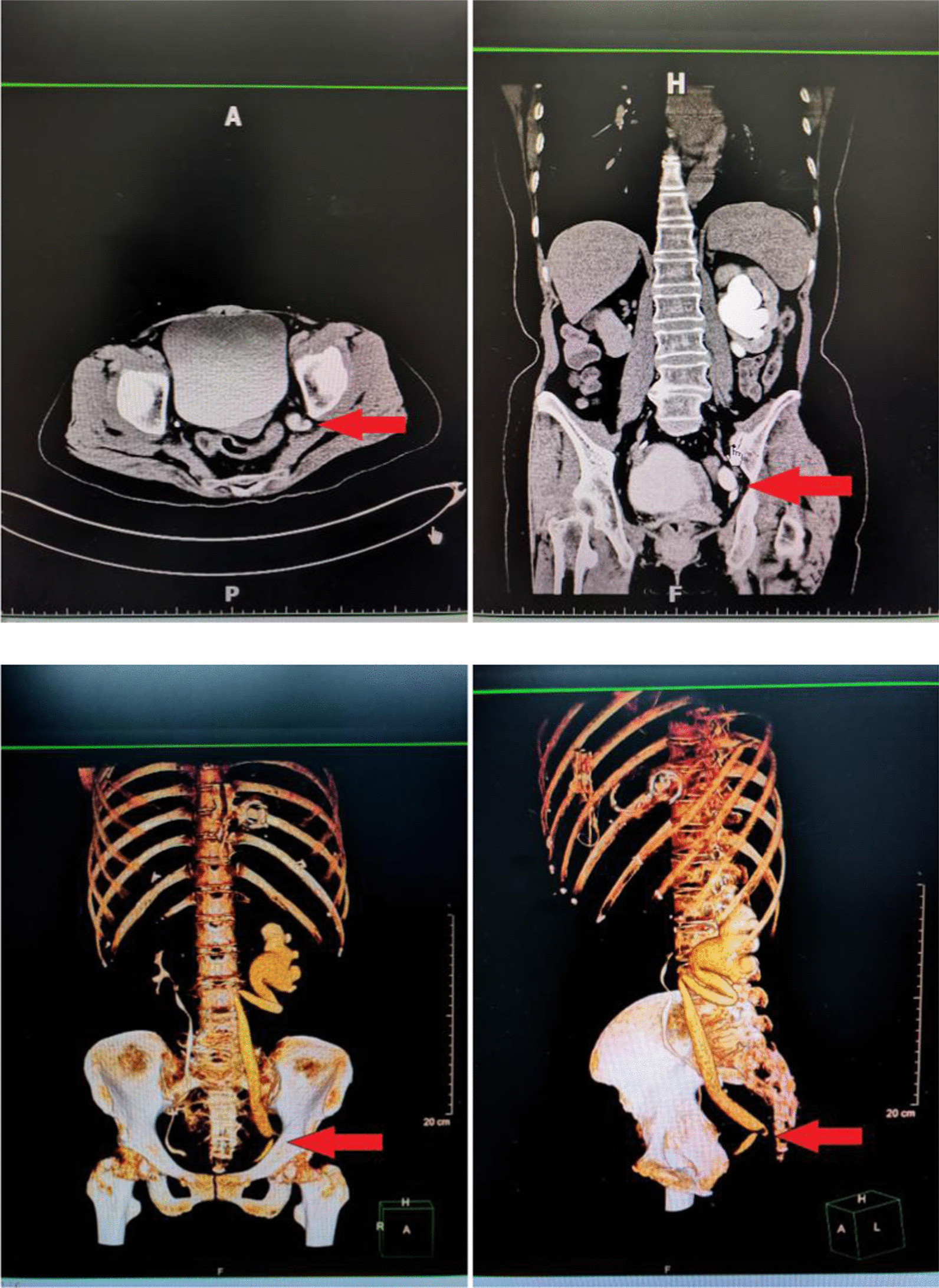
Urinary system CTU and three-dimensional reconstruction, suggesting tortuous hydronephrosis on the left ureter and hydronephrosis on the left

**Fig. 2:**
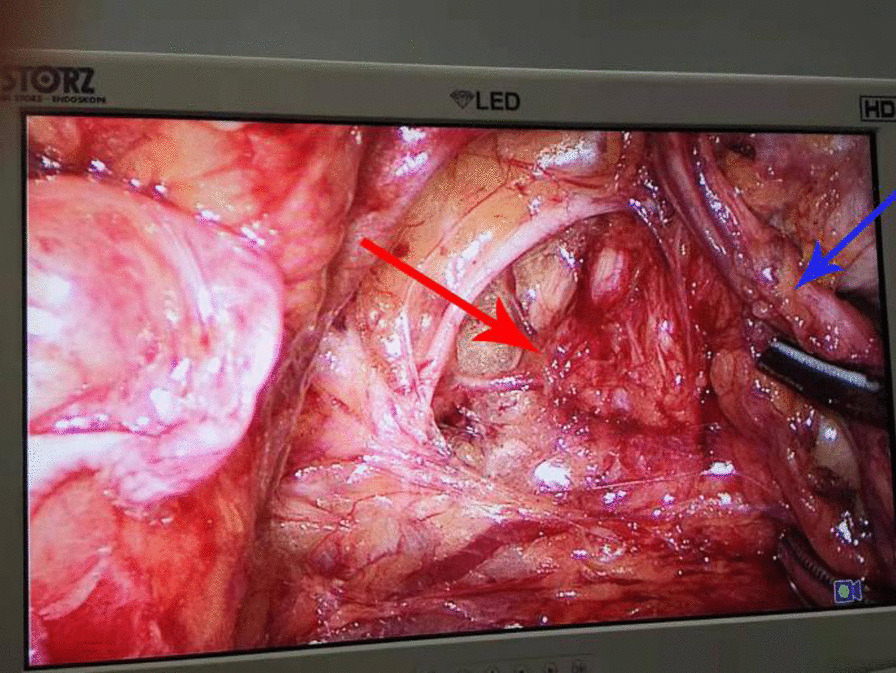
Intraoperative exploration revealed enlarged obturator. The red arrow refers to the enlarged obturator and the blue arrow refers to the twisted ureter

A 67-year-old female patient was admitted to another hospital for “rheumatic heart disease combined with mitral stenosis and regurgitation” and underwent “mitral valve replacement” surgery. During the hospitalization, a computerized tomography (CT) scan of the chest and abdomen was performed and it was found that the left kidney and ureter had hydrops. Three months later, in order to treat left kidney and ureteral hydrops, she was admitted to the Urology Department of our hospital. She has no symptoms such as frequent urination, urgency, abdominal pain, hematuria, and dysuria. She had no history of prior abdominal surgery, trauma, or congenital defects. Local examination showed no bulge or percussion pain in both kidney area and bilateral ureters. Laboratory examinations revealed a decrease in the glomerular filtration rate on both sides (70.15ml/min) and an increase in urine bacteria (7682.5/µL). After the physical examination, in order to further understand the degree of renal and ureteral hydrops, we recommended that she perform a computed tomography (CT) scan of the urinary tract. The results suggested that her left kidney and ureter were still present (Fig. 1). In order to clarify the cause of her kidney and ureteral hydrops, CTU imaging of the urinary tract was performed, and it was found that the left ureter pelvis herniated into the parietal pelvic fascia was accompanied by tortuosity and left hydronephrosis. In order to assess the function of the kidneys, she underwent nephro-dynamic imaging. The results suggest that the blood perfusion and function of the left kidney are slightly impaired, the excretion is significantly delayed, and the filtration rate of the left glomerulus is reduced (27.50ml/min); the glomerular filtration rate of the right kidney (38.60ml/min). The initial diagnosis was that the left ureter and hydronephrosis caused by pelvic perineal hernia, and general surgery consultation was invited, and laparoscopic exploration + tension-free repair of the left pelvic floor hernia was planned. We placed the patient in a lithotomy position, and placed trocars 1 cm above the navel, 5 cm on both sides of the navel, McBurney point and anti-McBurney point, and no hernias were found in the pelvic cavity through laparoscopy. Cut the left peritoneum during the operation and open the left side Toldt space.Toldt’s space is the fusion fascia space, which is formed by the fusion of the mesangium and the following tissues (mesenia or parietal peritoneum) after the midgut volvulus during embryonic development. It is called Toldt’s fascia, and the potential gap is called Toldt’s gap. Next, look for the left ureter, cut the peritoneum along the surface of the ureter to the pelvic floor. Later, observation of the pelvic cavity revealed that the left obturator space became larger, the ureter passed through the obturator area, and the upper ureter was significantly dilated (Fig. 2). Therefore, we changed the diagnosis to left obturator hernia with left ureteral hydrops and left hydronephrosis, and decided to perform left obturator hernia repair. And implemented the left ureterolysis, see the expansion of the ureter has returned to normal. The incised pelvic peritoneum was continuously sealed with a 3 -0 Barbed thread behind the ureter. Pelvic part of the ureter was moved into the abdominal cavity. No obvious bleeding was seen after flushing the abdominal cavity, and the incision was sutured layer by layer. The result of the operation was very successful. One week after the operation, she had a follow-up visit. Color Doppler ultrasound of the urinary system showed that the upper dilatation of the left ureter was better than before. And the patient is very satisfied with the treatment.

## Discussion

Ureteral obturator hernia is very rare in clinical practice. We only found 2 reports by searching and reviewing relevant literature (as of December 31, 2020, Pubmed, Embase and Cochrane, as well as CNKI and Wanfang electronic journals full-text database search).At present, the main cause of the disease is not clear. Some scholars believe that the main susceptibility factors may be piriformis atrophy, congenital or acquired defects of pelvic wall fascia [6]. Generally speaking, the clinical symptoms of ureteral obturator hernia are non-specific, including unclear abdominal pain, until the appearance of urinary obstructive diseases, such as renal insufficiency, urinary tract infection, kidney stones and uremia [7]. Weingarten, KE and others found a case of ureteral obturator hernia after kidney transplantation. This patient developed intermittent, dullness, and left lower abdominal discomfort within a few years after kidney transplantation. The serum creatinine level rose from 133 to 293 µmol/L. L (1.5 mg/dL to 3.3 mg/dL). In the next 2 weeks, the patient’s renal function deteriorated, and the serum creatinine level showed an irregular upward trend, with a peak value of 506 µmol/L (5.7 mg/dL). Ultrasound examination of the transplanted kidney showed moderate hydronephrosis, and antegrade pyelography showed a long and tortuous ureter, accompanied by distal ureteral obstruction. So they first placed a percutaneous nephrostomy tube for drainage, and manually reset the prolapsed ureter through gentle palpation and groin manipulation. Subsequently, obturator hernia repair was performed  [8]. Izzo, M. et al. reported a case of obturator hernia with ureteral compression. This patient suddenly developed vomiting, weakness, constipation and left lower abdominal pain and came to see a doctor. After hospitalization, he found C-reactive protein and creatinine levels. High; Urine examination showed leukopenia and hematuria, ultrasound examination showed hydronephrosis in the left kidney, and computed tomography showed that the left obturator foramen and ipsilateral ureter were compressed, and the small intestinal ring was completely prominent. Because the patient’s renal function was damaged, they considered elective surgery after the renal function was restored  [9].

The clinical symptoms of ureteral obturator hernia are non-specific. In rare cases, just like our case, there are no symptoms, only the preoperative CT suggests that the lower part of the ureter is obstructed. It is difficult to diagnose ureteral obturator hernia by physical examination alone. In essence, it is idiopathic ureteral stenosis. Therefore, proper assessment and treatment of ureteral stenosis is of great significance for protecting renal function and eliminating malignant tumors. The causes of ureteral stenosis include: (A) Malignant diseases, such as transitional cell carcinoma and cervical tumors. In addition, ureteral stenosis can also occur in the in situ metastasis of cancers such as cervical cancer, prostate cancer, and ovarian cancer. (B) Ureteral stones. (C) Radiation injury. (D) Ischemia and trauma caused by surgery. (E) Injury caused by ureteroscopy. (F) Infection, such as urinary tuberculosis. Therefore, urography (i.e., intravenous urography, retrograde pyelography and CT) can assist in the diagnosis [10]. Urography can identify the location of the obstruction. The main imaging manifestations are ureteral dilatation, hydronephrosis, and even ureteral cyst. Frank C. Lin et al. suggested that ureteral hernia should be treated conservatively unless there is emergency treatment (such as obstruction, uremia, or intractable lumbar pain). And conduct long-term laboratory and imaging follow-up to monitor progress [3].

We believe that when ureteral hernia leads to ureteral obstruction and impaired renal function, intervention should be made as soon as possible. Especially in this type of patient, long-term hydronephrosis may lead to kidney damage and infection, and even eventually Will cause the kidneys to fail [11]. When renal function is severely impaired, percutaneous nephrostomy for external drainage and ureteral stent implantation for internal drainage will help reduce obstructive symptoms and protect renal function. When the protruding ureter appears obvious dilation, atresia, inflammation or necrosis, the ureter should be removed, and then the ureterocystoscope should be performed [7]. There are many surgical approaches, including inguinal approach, retropubic approach, and transperitoneal approach. Generally, the abdominal approach via a low median incision is the most popular because it can fully expose the obturator ring and identify and remove any adhesions. This method allows the surgeon to establish a diagnosis, avoid any obturator vessels, and better expose the obturator ring [9].

Once unexplained hydronephrosis or ureteral obstruction is found, you need to be alert to the possibility of ureteral hernia. Early urography can improve the possibility of accurate diagnosis. When a patient has impaired renal function, early surgical intervention is effective.

## Data Availability

All data generated or analysed during this study are included in this published article.
